# Recovery of Metals from Electronic Waste-Printed Circuit Boards by Ionic Liquids, DESs and Organophosphorous-Based Acid Extraction

**DOI:** 10.3390/molecules27154984

**Published:** 2022-08-05

**Authors:** Aneta Łukomska, Anna Wiśniewska, Zbigniew Dąbrowski, Jakub Lach, Kamil Wróbel, Dorota Kolasa, Urszula Domańska

**Affiliations:** ŁUKASIEWICZ Research Network–Industrial Chemistry Institute, Rydygiera 8 Str., 01-793 Warsaw, Poland

**Keywords:** metal extraction from electronic waste, ionic liquids, DESs, organophosphorous-based acid, extraction efficiency

## Abstract

The extraction of metals from waste printed circuit boards (WPCBs) with ionic liquids (ILs), Deep Eutectic Solvents (DESs) and organophosphorous-based acid (Cyanex 272) has been presented. The study was undertaken to assess the effectiveness of the application of the new leaching liquids, and the new method of extraction of metals from the leachate and the solid phase with or without the leaching process. Solvent extraction from the liquid leachate phase has been studied in detail with popular ILs, such as tetraoctylphosphonium bromide, {[P_8,8,8,8_][Br] and tributyltetradecylphosphonium chloride, [P_4,4,4,14_][Cl] using Aqueous Biphasic Systems (ABS) method. Trihexyltetradecylphosphonium bis(2,4,4-trimethylpentyl) phosphinate, [P_6,6,6,14_][Cyanex272], ([P_6,6,6,14_][BTMPP]), trihexyltetradecylphosphonium thiocyanate, [P_6,6,6,14_][SCN], methyltrioctylammonium chloride (Aliquat 336), as well as bis(2,4,4-trimethylpentyl)phosphinic acid (Cyanex 272) were also used in the extraction of metals from the leachate. Two DESs (1) {choline chloride + lactic acid, 1:2} and (2) {choline chloride + malonic acid, 1:1} were used in the extraction of metals from the solid phase. The extraction behavior of metals with DESs was compared with that performed with three new bi-functional ILs: didecyldimethylammonium salicylate, [N_10,10,1,1_][Sal], didecyldimethylammonium bis(2-ethylhexyl) phosphate, [N_10,10,1,1_][D2EHPA], and didecyldimethylammonium bis(2,4,4-trimethylpentyl) phosphinate, [N_10,10,1,1_][Cyanex272]. The [P_6,6,6,14_][Cyanex272]/toluene and (Cyanex 272 + diethyl phosphite ester) mixtures exhibited a high extraction efficiency of about 50–90% for different metal ions from the leachate. High extraction efficiency of about 90–100 wt% with the ABS method using the mixture {[P_8,8,8,8_][Br], or [P_4,4,4,14_][Cl] + NaCl + H_2_O_2_ + post-leaching liquid phase} was obtained. The DES 2 revealed the efficiency of copper extraction, *E*_Cu_ = 15.8 wt% and silver, *E*_Ag_ = 20.1 wt% at pH = 5 from the solid phase after the thermal pre-treatment and acid leaching. The solid phase extraction efficiency after thermal pre-treatment only was (*E*_Cu_ = 9.6 wt% and *E*_Ag_ = 14.2 wt%). The use of new bi-functional ILs did not improve the efficiency of the extraction of metal ions from the solid phase. Process factors such as solvent concentration, extraction additives, stripping and leaching methods, temperature, pH and liquid/solid as well as organic/water ratios were under control. For all the systems, the selectivity and distribution ratios were described. The proposed extraction processes can represent alternative paths in new technologies for recovering metals from electronic secondary waste.

## 1. Introduction

The recovery of valuable metals from waste printed circuit boards, WPCBs is traditionally carried out using hydrometallurgy and pyrometallurgy with primary leaching with aqua regia, or other acids [1,2,3]. Electro-waste contains large amounts of valuable metals, such as copper, silver, palladium, nickel, aluminium, zinc, gold, iron, lead, and others [1,2,3]. It is well known that annually over 70 million tons of e-waste from various technological processes should be recycled based on pyrometallurgy, hydrometallurgy or solvometallurgy. In hydrometallurgical processes, acid and alkaline leaching is generally employed to dissolve metals such as Cu, Au, Ag, and Pd from the spent e-waste [1,2,3]. Recent studies have proposed extraction with ionic liquids (ILs) by liquid-liquid extraction (LLE) and electrodeposition [4]. Bi-functional ILs have been used for the extraction of Mo and V from spent petroleum catalysts [5]. As an example, the IL was constructed from IL methyltrioctylammonium chloride, [N_8,8,8,1_][Cl], (Aliquat 336) and bis(2-ethylhexyl)hydrogen phosphate, D2EHPA as a very active cationic-anionic extractant of metals in the presence of H_2_O_2_ [5].

A review of metal extraction with ILs (especially with bis{(trifluoromethyl)sulfonyl}imide, [NTf_2_]^-^anion), using Deep Eutectic Solvents (DESs) with the addition of crown ethers, amines and other substances, as well as using organophosphorus-based acids has been presented [6,7,8].

Copper extraction at the level of 72 wt% from the “black mass” of waste Li-ion batteries was obtained using trihexyltetradecylphosphonium thiocyanate, [P_6,6,6,14_][SCN] with the addition of H_2_O_2_ and didecyldimethylammonium chloride surfactant, DDACl, as well as using Cyanex 272 with the addition of diethyl phosphite ester [9]. Extraction of copper from solid e-waste with hydrogen sulphate ammonium and imidazolium-based ILs, such as [N_1,1,8,H_][HSO_4_] with the addition of H_2_O_2_ at *T* = 348 K (1 h) was obtained at the level of 20–33 wt% [10]. 98% Extraction of copper from the solid phase was obtained with the use of bisulphate ILs: [BMIM][HSO_4_], [PS-MIM][HSO_4_], [CM-MIM][HSO_4_], [di-Ac-IM][HSO_4_], [C_6_(di-Ac-IM)[HSO_4_]) with the addition of H_2_O_2_, after preliminary leaching with 1-butyl-3-methylimidazolium tetrafluoroborate, [BMIM][BF_4_] [11]. Extraction of copper, silver and gold from WPCBs with 1-butyl-3-methylimidazolium bis{(trifluoromethyl)sulfonyl}imide, [BMIM][NTf_2_], 1-butyl-3-methylimidazolium hexafluorophosphate, [BMIM][PF_6_] and Cyphos 101 ([P_6,6,6,14_][Cl]) after preliminary leaching with (H_2_SO_4_ + H_2_O_2_) and then (35% HCl + 55% HNO_3_) was obtained at the of level 90% and 99% for Cu at the temperature *T* = 343 K, pH = 1 with a solid to liquid ratio of 1:1 [12]. The aqueous solution contained the following species of metals: AgCl, CuCl_2_, CuCl^+^ and CuCl_4_^2−^ [12].

The use of ILs in hydrometallurgy has been popular for many years [13]. The extraction of many different metals from various materials with ILs is well described [14,15,16,17]. The popular IL, used for the extraction of metals from petroleum catalysts waste and e-waste is 1-butyl-3-methylimidazolium hydrogen sulphate, [BMIM][HSO_4_] [18]. It is a non-toxic, easily synthesized IL with the possibility of recirculation [18]. [BMIM][HSO_4_] was also used instead of H_2_SO_4_ in thiourea for Au extraction with the use of oxygenating agents such as Fe_2_O_3_, H_2_O_2_, or KHSO_5_ [15,16]. The hydrogen sulphate anion was used with different cations such as [HMIM]^+^, [OMIM]^+^ and [EMIM]^+^ [19,20]. IL [BMIM][HSO_4_] was used for the extraction of Cu (82%) and Zn (99%) from brass waste with oxidizing agents such as H_2_O_2_ and KHSO_5_ at room temperature using the ratio IL:A = 1:1 *v/v* [14,21]. IL 1-ethyl-3-methylimidazolium hydrogen sulphate, [EMIM][HSO_4_] was used for the extraction of metals Fe (80%), Sc (68%), Ti (60%) and Al (20%) from boxes at high temperature [22]. In general, many ILs were used for the extraction of metals in acidic solutions [23,24], as well as bi-functional ILs extractants used at a specified pH with the addition of Na_2_SO_4,_ NaCl, or NaNO_3_ [24]. ILs with ammonium, or phosphonium cations and different functionalized anions such as thiol-, thioether-, hydroxyl-, carboxylate- and thiocyanate have been used for the extraction of metals from communal “wastewater” with a high extraction efficiency of 95% for Ag, Cu, Hg and Pt [25]. IL [BMIM][NTf_2_] was found to be a good agent for the extraction of Au from chlorinated aqueous solutions [26]. Phosphonium IL, Cyphos 101 was used with good results for the extraction of many metals including Au(III) from aqueous solutions with HCl [23,27,28]. The technique of biopolymer capsules was used for the extraction of Au(III) from aqueous solutions with HCl [28]. The use of tributylmethylammonium chloride, [N_4,4,4,1_][Cl] with the addition of trichloroizocyanic acid (TCCA) showed 100% extraction of metals: Au, Pd, Cu, and Ag at low temperature *T* = 298 K [29].

One of the first pieces of information about the extraction of Cu, Zn and Al from solid WPCBs material was presented with [BMIM][HSO_4_] and H_2_O_2_ at the temperature *T* = 343 K for 2 h [30]. The extraction of Cu (100%) was carried out from a solution of 25 cm^3^ 80% IL *v/v* and 10 cm^3^ 30% H_2_O_2_ in a ratio of solid:liquid = 1:25 [30].

The recycling processes of Au, Ag, Pd, and Pt from WPCBs with the use of glycine and sodium cyanide were presented in [31], as well as Cu [32]. However, a very inconvenient solid to liquid phase ratio of of 1:100 was used with the addition of 10% H_2_O_2_ at the temperature *T* = 303 K (2 h), pH = 6–6.5 [32]. The Cu(II) extraction efficiency was 94%. Glycine is the simplest environmentally friendly amino acid and is used in heteronuclear complexes with metal ions.

In this work, the extraction of metals from WPCBs after thermal pretreatment at the temperature *T* = 1023 K for 7 h and different acid leaching procedures with ionic liquids (ILs), DESs and Cyanex 272 have been proposed.

Liquid-liquid extraction from the liquid and solid phases with various extraction solvents, with the addition of hydrogen peroxide (H_2_O_2_) and didecyldimethylammonium chloride surfactant, [N_10,10,1,1_][Cl], DDACl (50 wt%) for the extraction from the solid phase, is presented. The ILs were used in Aqueous Biphasic System (ABS) for the extraction of metals from aqueous leachate solutions with ILs {[P_8,8,8,8_][Br], or [P_4,4,4,14_][Cl] + NaCl + liquid leachate phase}.

The possibility of extracting and separating metal ions from the solid phase (leached, or with the elimination of acidic leaching at high temperatures) was investigated using popular DESs {choline chloride + lactic acid, 1:2} and {choline chloride + malonic acid, 1:1} and three new, synthesized bi-functional ILs: didecyldimethylammonium salicylate, [N_10,10,1,1_][Sal], C_29_H_53_NO_3_, didecyldimethylammonium bis(2-ethylhexyl)phosphate [N_10,10,1,1_][D2EHPA], C_38_H_82_NO_4_P, and didecyldimethylammonium bis(2,4,4-trimethylpentyl)phosphinate [N_10,10,1,1_][Cyanex272], C_38_H_82_NO_2_P.

The results of this research may provide a different ecological and efficient approach for Cu and Ag extraction from WPCBs at different costs.

## 2. Results and Discussion

### 2.1. Analysis of the Solid WPCBs Samples and Post-Leaching Solutions

**Figure 1:**
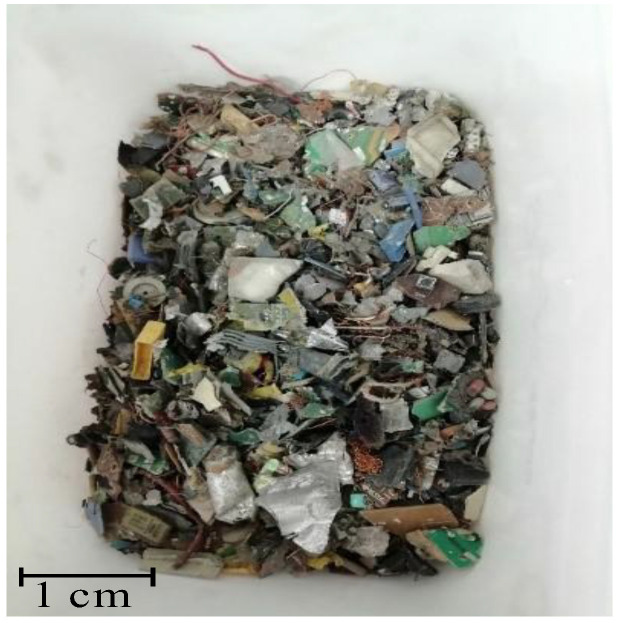
Solid WPCBs starting material.

**Figure 2:**
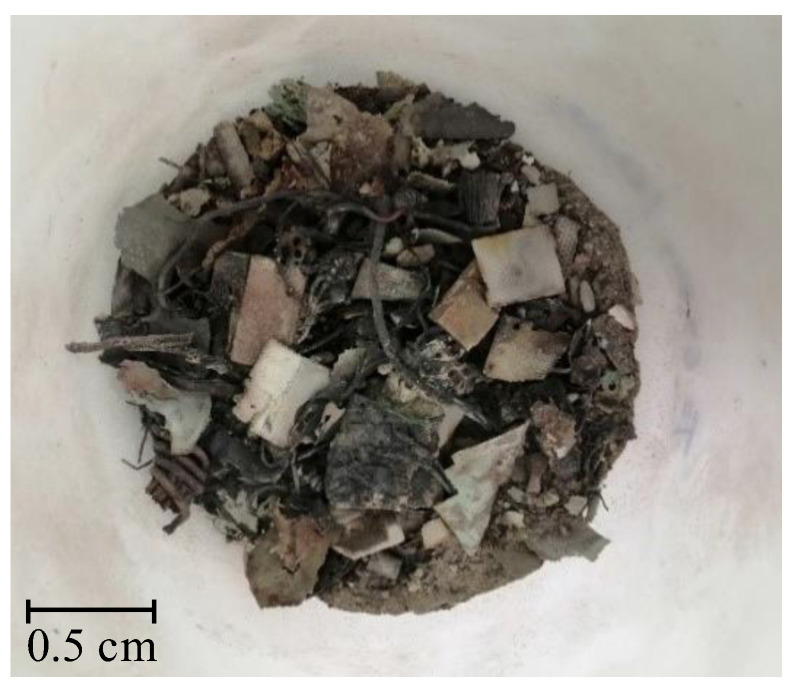
Solid WPCBs material after thermal pre-treatment at the temperature *T* = 1023 K for 7 h.

**Figure 3:**
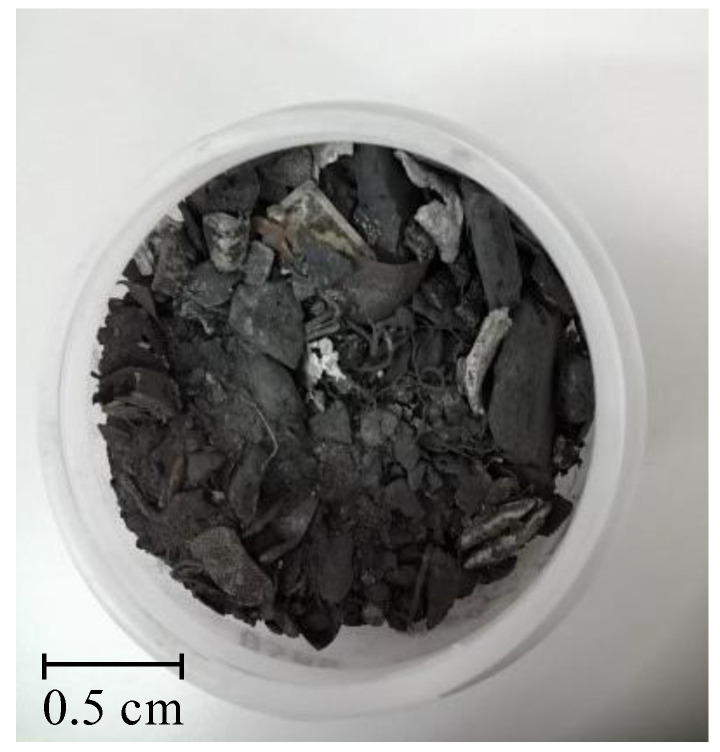
Solid WPCBs material after thermal pre-treatment and the I leaching (4M H_2_SO_4_ + 100 g/dm^3^ (NH_2_)_2_CS + 13 g/dm^3^ Fe_2_(SO_4_)_3_).

**Figure 4:**
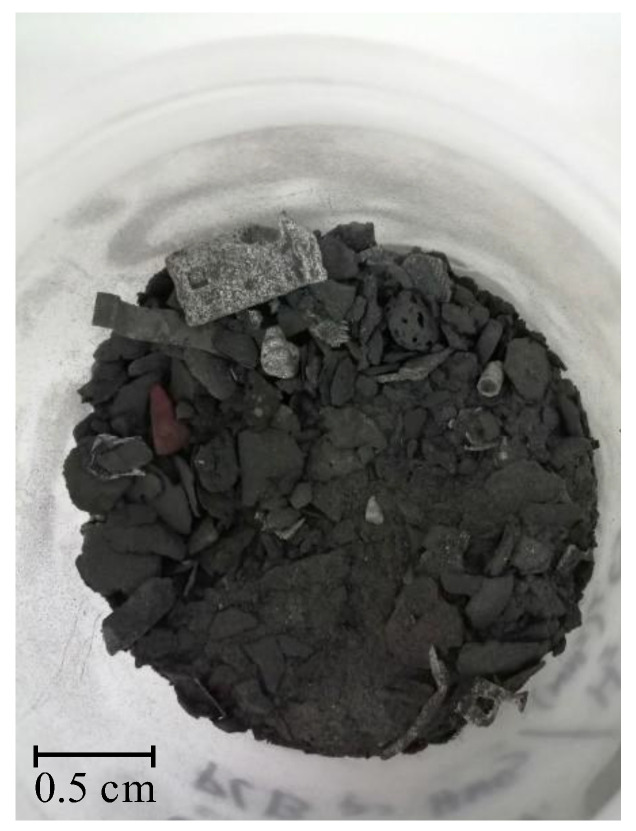
Solid WPCBs material after thermal pre-treatment and two steps of the II leaching with 5M HNO_3_ and (4M H_2_SO_4_ + 100 g/dm^3^ (NH_2_)_2_CS + 13 g/dm^3^ Fe_2_(SO_4_)_3_), respectively.

Table 1 summarizes the results of the metal content in the solid WPCBs samples of the starting material (see Figure 1) and the material after thermal pre-treatment (see Figure 2) and then the I and II leaching processes (see Figure 3 and Figure 4). Table 1 shows that after the I leaching, the copper concentration decreased from 335 g/kg to 235 g/kg and the mass of the solid phase decreased from 0.670 kg to 0.575 kg. It is clear from this result that part of the copper has passed into the liquid phase.

The amounts of metals leached into the liquid phase after the I and II leaching processes are presented in Table 2. The results of the solid phase sample analysis for metal content after alkalization of the post-leaching solutions to pH = 3, for the I liquid leachate phase, for the II liquid leachate phase (first step), and for the II liquid leachate phase (second step) are listed in Table 3. Table 4 presents the results of the liquid phase samples analysis for metal content in the liquid phase after alkalization of the post-leaching solutions to pH = 3.

The concentration of Pd and Au in the solid phase after leaching was below the detection limit of the FAAS method and is not listed in Table 1. The Sn content was not measured at all by FAAS in the solid samples. This element has not been the main focus of metal recovery research. The ICP-OES analysis has shown more metals in the liquid phase samples after the leaching process (see Table 2).

Table 1 shows that after the I leaching, Cu(II) passed into the liquid phase from 335 g/kg (solid material after the thermal pre-treatment) to 235 g/kg. The other metals for the most part remained in the solid phase. After the II leaching (second step), a much greater transfer of Ag(I) and other metals (except Al and Fe) to the liquid phase was observed, and the Ag(I) content in the solid material changed from 721 mg/kg to almost 0 mg/kg and Cu(II) from 335 g/kg to almost 0 g/kg. The results in Table 1 were calculated taking into account the mass changes of the samples after the thermal pre-treatment and all steps of leaching. As can be seen from Table 1, the further extraction of metals from the solid material after the II leaching (second step) does not have the essential meaning as this solid material does not contain important metals such as copper or silver. The results of the ICP-MS analysis of the liquid phase samples for the metal content after the leaching processes, revealed 29544 mg/kg of Cu(II), 6985 mg/kg of Fe(II), 6110 mg/kg of Al(III), 2205 mg/kg of Zn (II) and 1194 mg/kg of Pb(II) after the II leaching (first step) (see Table 2).

After this procedure, the post-leaching solutions were treated with NaOH (solid) until pH = 3 for the precipitation of iron and aluminum hydroxides. The obtained grey and white solid phases were analyzed by the SEM/EDS method (see Table 3) and the other liquid phases were analyzed by the ICP-OES technique (see Table 4).

**Figure 5:**
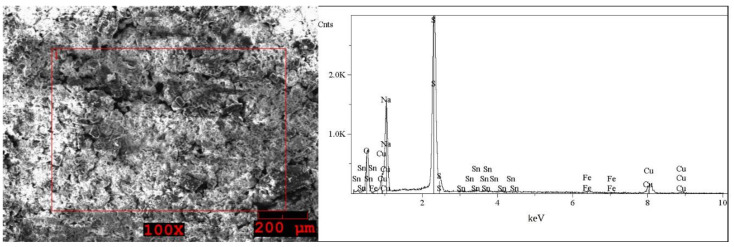
SEM image of the grey solid phase sample precipitated out from the post-leaching solution I and EDS spectrum taken from the area marked in the image.

**Figure 6:**
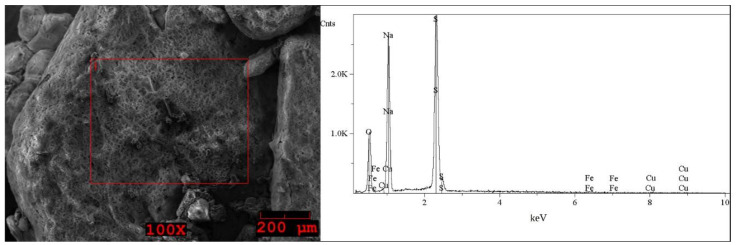
SEM image of the white solid phase sample precipitated out from the post-leaching solution I and EDS spectrum taken from the area marked in the image.

**Figure 7:**
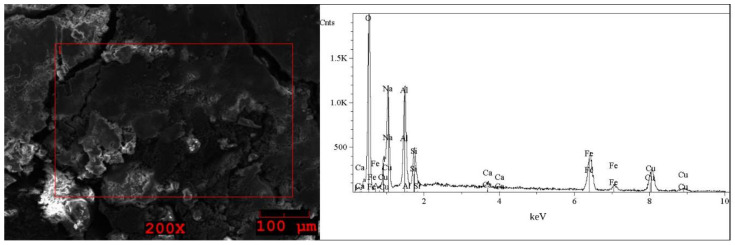
SEM image of the brown solid phase sample precipitated out from the post-leaching solution II (first step) and EDS spectrum taken from the area marked in the image.

**Figure 8:**
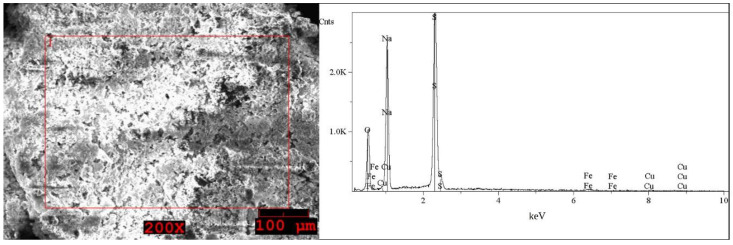
SEM image of the grey solid phase sample precipitated out from the post-leaching solution II (second step) and EDS spectrum taken from the area marked in the image.

**Figure 9:**
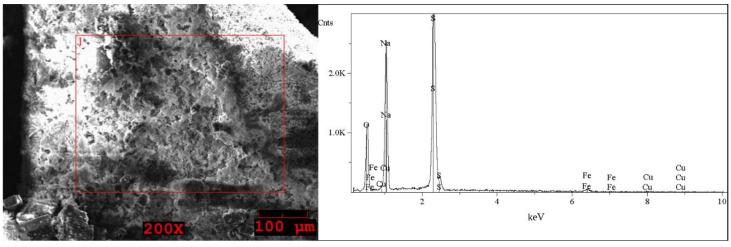
SEM image of the white solid phase sample precipitated out from the post-leaching solution II (second step) and EDS spectrum taken from the area marked in the image.

The SEM images and EDS spectra, taken from micro-areas of the solid samples are shown in Figure 5, Figure 6, Figure 7, Figure 8 and Figure 9. Irregular shapes and agglomerates of grains were observed in the deposited films. As shown in Figure 5, for the grey solid phase obtained from the liquid leachate phase I, the coexistence of oxygen (O), sodium (Na), sulfur (S), iron (Fe), copper (Cu) and tin (Sn) is observed on the surface. In the EDS spectra (Figure 6) taken from micro-areas of the white solid phase obtained from the liquid leachate phase I, such elements as oxygen (O), sodium (Na), sulfur (S), iron (Fe) and copper (Cu) were identified on the surface. For the brown solid phase sample, stripped from the liquid leachate phase II (first step) (Figure 7) the SEM/EDS analysis showed the presence of oxygen (O), sodium (Na), aluminum (Al), silicon (Si), iron (Fe), copper (Cu) and calcium (Ca) on the surface.

As a result of the semi-quantitative analysis by the SEM/EDS method, it was found that the obtained solid phases contained mainly the above-mentioned elements, such as e.g., copper (7–11 wt%) or iron (9–11 wt%) in the brown solid phase precipitated out from the post-leaching solution II (first step) (see Table 3). Table 4 presents the results of the ICP-OES analysis for the metal content in the liquid phases at pH = 3 after the leaching processes I and II.

### 2.2. Extraction with Ionic Liquids and Organophosphorus Based Acid from the Post-Leaching Solutions

In this study, the IL was used to leach various metals from WPCBs after the process of thermal pre-treatment at the temperature *T* = 1023 K for 7 h and acid leaching as described above. All ILs used in this work are listed in Table 5 and the other chemicals in Table 6.

The extraction efficiency (*E*) and distribution ratio (*D*) were determined using the following equations:

*E* (wt%) = 100 × (m_E,O_/m_0_) (1)

(2) $$D=\frac{V_{A}}{V_{O}}\times (m_{E,O}/m_{0})$$

where m_0_ (g) is the initial metal content in the solid starting material or post-leaching solution, m_E,O_ (g) is the content of metal ions in the organic phase after extraction and stripping; *V_A_* and *V_O_* (cm^3^) are the volume of the aqueous phase and the organic phase, respectively.

After leaching, metal like copper can pass into the aqueous phase as a Cu^2+^ cation and/or as an [Cu(SO_4_)_2_]^2−^ anion in an aqueous solution of H_2_SO_4_, or [CuCl_4_]^2−^ with Cl^−^ anions from DDACl in the aqueous phase.

Unfortunately, popular for the extraction of metals IL-Aliquat 336 presented the extraction efficiency for all metals only at the level of 0.1–5 wt% for the post-leaching solution I at pH = 3 and pH = 6. The [P_6,6,6,14_][SCN] showed even worst results, about 0.1–1.2 wt% for all metals at pH = 5.5.

The obtained next results with various ILs showed that [P_6,6,6,14_][Cyanex272], ([P_6,6,6,14_][BTMPP])/toluene at pH = 7 and (Cyanex 272 + diethyl phosphite ester) at pH = 3 can successfully leach metals out, with recovery close to 90–95 wt%, *D*_Al_ = 0.91, *D*_Zn_ = 0.96 for Al(III) and Zn(II) ([P_6,6,6,14_][Cyanex272]/toluene), and 100 wt%, *D*_Ag_ = 1 for Ag(I) (Cyanex 272 + ester). The extraction efficiency for Cu(II) was *E*_Cu_ = 59.3 wt%, *D*_Cu_ = 0.59 and for Ag(I) *E*_Ag_ = 66.7 wt%, *D*_Ag_ = 0.67 with [P_6,6,6,14_][Cyanex272]/toluene at pH = 7. Quite high the extraction efficiency was also observed for Zn(II), *E*_Zn_ = 51.7 wt%, *D*_Zn_ = 0.52 with (Cyanex 272 + ester) at pH = 3. Complete results are listed in Table 7. As shown in Table 7, all metals were not quantitatively extracted in one extraction step, meaning that there is a need for a scrubbing step with different analytical methods for different metals after the extraction process.

The mechanism for the metal extraction from the aqueous solution to the IL phase is proposed as an “ion exchange reaction”. The metal cation has to replace the IL cation, which moves to the aqueous phase [33]. In general, the reaction with IL is as follows:

Me^2+^_(A)_ + 2 [Cation]^+^[Anion]^−^_(O)_ = Me[Anion]_2(O)_ + 2 [Cation]^+^_(A)_ (3)

The mechanism of metal extraction with Cyanex 272 may be as follow [34]:

4 HA_(org)_ + Me^2+^_(A)_ = MeA_2_·2 HA_(O)_ + 2 H^+^_(A)_ (4)

The leaching time, IL concentration, hydrogen peroxide addition, aqueous to organic phase (1:1) and temperature were similar in all experiments. The results obtained with Aliquat 336 and [P_6,6,6,14_][SCN] with the addition of hydrogen peroxide in the liquid phase were not successful.

### 2.3. Extraction with ILs from the Post-Leaching Solutions Using ABS Method

ABS is a popular method of extracting metal ions from aqueous solutions [35,36,37]. Two liquid systems were used for the separation of metal ions from the liquid phase at pH = 3:{[P_8,8,8,8_][Br] + NaCl + H_2_O_2_ + liquid leachate phase I or II} and {[P_4,4,4,14_][Cl] + NaCl + H_2_O_2_ + liquid leachate phase, I or II}. The extraction efficiency was calculated from the concentration of metal ions in the organic phase. The results are presented in Table 8. The copper was extracted to the IL phase with the extraction efficiency of 96.4 wt%–100.0 wt% and distribution ratio, *D*_Cu_ = 0.96–1, and silver with the extraction efficiency of 88.9 wt%–100.0 wt%, *D*_Ag_ = 0.90–1 using two ILs with the addition of H_2_O_2_. Satisfied results with both ILs were also obtained for Fe(II), *E*_Fe_ = 82.6 wt%–94.1 wt%, *D*_Zn_ = 0.82–0.94 excluding the system {[P_4,4,4,14_][Cl] + NaCl + H_2_O_2_ + liquid leachate phase II (first step)}, *E*_Fe_ = 45.5 wt%, *D*_Fe_ = 0.45. Similar very good results were obtained for Zn(II) with the extraction efficiency of *E*_Zn_ = 99.5 wt%–100.0 wt% and distribution ratio *D*_Zn_ = 0.99–1. Only Al(III) after extraction was transferred mainly to the aqueous phase with the extraction efficiency of *E*_Al_ = 78.2 wt%–99.7 wt%. Unfortunately, all metals were transferred together to the organic phase (excluding Al(III)). No quantitative extraction of the various metal ions to the organic, or aqueous phase was observed. The process of extraction from the post-leaching solutions using the ABS method is more attractive and the results are much better than those, obtained with ILs. The results presented in Table 8 confirmed that the ABS method is able to extract metal ions from the liquid leachate phases with high extraction efficiency in the presence of H_2_O_2_. One example of extraction without H_2_O_2_ in the system {[P_8,8,8,8_][Br] + NaCl + liquid leachate phase II (first step)} revealed much lower extraction efficiencies for all metals.

H_2_O_2_ can oxidize metallic Cu from a solid material to Cu ions in the liquid phase. Increasing the temperature increases the extraction efficiency, but only up to the temperature *T* = 323 K. At a higher temperature, H_2_O_2_ would be decomposed. Over the temperature *T* = 323 K decomposition of H_2_O_2_ dominates and the efficiency of Cu leaching decreases. According to reaction (5), the ions (for example Cu^2+^) enter the solution [32].

Cu + H_2_O_2_ + 2 H^+^ = Cu^2+^ + 2 H_2_O (5)

Typical leaching of Cu from WPCBs with sulfuric acid (or other acids) in the presence of H_2_O_2_ runs according to the following reaction:

Cu + H_2_O_2_ + H_2_SO_4_ = CuSO_4_ + 2 H_2_O (6)

In the ABS method, the salt-out reagent, NaCl helps to increase the effective activity of metal ions in the solution. Under the acidic conditions used, M^2+^ metal ions and MeCl_2_ complexes (coming from NaCl and the IL) are in the solution [34].

### 2.4. Extraction with DESs and bi-Functional ILs from the Solid WPCB Samples

In recent years DESs have been used as primary extracting solvents for the extraction of metal ions from the solid material of spent batteries [9]. The advantage of using DESs in place of the ILs is their lower cost (especially those based on choline chloride) and less corrosive effects on the equipment.

In this work, the solid material after thermal pre-treatment at the temperature *T* = 1023 K for 7 h and acid leaching is used directly in the DES solution with the addition of DDACl and H_2_O_2_. The solid WPCBs sample was stirred with the solution of (DES + H_2_O + DDACl + H_2_O_2_) for 2 h at the temperature *T* = 333 K. Two popular DESs were used for the extraction of metal ions: DES 1 (choline chloride:lactic acid, 1:2) and DES 2 (choline chloride:malonic acid, 1:1). After phase separation in the mixtures, the content of metal ions were analyzed. The results of the extraction of metals from the solid material after the I leaching with {4M H_2_SO_4_ + 100 g/dm^3^ (NH_2_)_2_CS + 13 g/dm^3^ Fe_2_(SO_4_)_3_} are presented in Table 9. Unfortunately in the case of DES 1, the high extraction efficiency was observed at pH = 2.5 only for Al(III), *E*_Al_ = 51.2 wt% with distribution ratio *D*_Al_ = 0.51, and for Fe(II) *E*_Fe_ = 27.7 wt% with *D*_Fe_ = 0.28. Better results were obtained using DES 2, where the extraction efficiency for Cu(II) and Ag(I) was *E*_Cu_ = 8.8 wt%, *D*_Cu_ = 0.09 and *E*_Ag_ = 26.4 wt%, *D*_Ag_ = 0.26, respectively. Much higher extraction efficiency was found for Al(III) with DES 2: *E*_Al_ = 91.5 wt%, *D*_Al_ = 0.91 and *E*_Fe_ = 19.7 wt%, *D*_Fe_ = 0.20 for Fe(II). Better results for important metals were obtained at pH = 5 with DES 1. The extraction efficiency for Al(III), was only *E*_Al_ = 52.7 wt%, *D*_Al_ = 0.53 and *E*_Fe_ = 24.0 wt%, *D*_Fe_ = 0.24 for Fe(II). The changing of pH increased the extraction efficiency for silver up to *E*_Ag_ = 13.0 wt%, *D*_Ag_ = 0.13. Better results for copper were obtained using DES 2, where the extractions of Cu(II) and Ag(I) were *E*_Cu_ = 15.8 wt%, *D*_Cu_ = 0.16 and *E*_Ag_ = 20.1 wt%, *D*_Ag_ = 0.20, respectively. Lower extraction efficiency was shown for Al(III) with DES 2, *E*_Al_ = 48.9 wt%, *D*_Al_ = 0.49 and for Fe(II), *E*_Fe_ = 24.7 wt%, *D*_Fe_ = 0.25. Thus, the extraction of Al(III) decreased at pH = 5 and the extraction of Cu(II) increased with DES 2 at pH = 5.

Three new bi-functional ILs were used for the extraction of metal ions for comparison with DESs. The results of the extraction of metals from the solid material after thermal pre-treatment (*T* = 1023 K, 7 h) and after the I leaching with {4M H_2_SO_4_ + 100 g/dm^3^ (NH_2_)_2_CS + 13 g/dm^3^ Fe_2_(SO_4_)_3_} are presented in Table 9, and for the solid WPCBs sample after thermal pre-treatment (*T* = 1023 K, 7 h) without leaching in Table 10. High efficiency of metal extraction from the solid material after the I leaching with {4M H_2_SO_4_ + 100 g/dm^3^ (NH_2_)_2_CS + 13 g/dm^3^ Fe_2_(SO_4_)_3_} was observed for Al(III) with [N_10,10,1,1_][Sal], *E*_Al_ = 25.5 wt%, *D*_Al_ = 0.25. [N_10,10,1,1_][Sal] also extracted Cu(II) and Fe(II), *E*_Cu_ = 9.2 wt%, *D*_cu_ = 0.09 and *E*_Fe_ = 7.8 wt%, *D*_Fe_ = 0.08. The [N_10,10,1,1_][D2EHPA] and [N_10,10,1,1_][Cyanex272] bi-functional ILs were not effective in the extraction.

DES 2 and DES 2 with the addition of Na_2_SO_4_ were used for the extraction at pH = 5 from the solid material after the process of thermal pre-treatment at the temperature *T* = 1023 K for 7 h. The addition of Na_2_SO_4_ did not improve the extraction. The results for DES 2 (without Na_2_SO_4_) were as follows: for Al(III), *E*_Al_ = 67.3 wt%, *D*_Al_ = 0.67, for Cu(II), *E*_Cu_ = 9.6 wt%, *D*_Cu_ = 0.09, and for Ag(I), *E*_Ag_ = 14.2 wt%, *D*_Ag_= 0.14 (see Table 10). The results of extraction with DES 2 at pH = 5 are slightly worse than those obtained for the solid material after leaching (*E*_Cu_ = 15.8 wt% and *E*_Ag_ = 20.1 wt%).

The mechanism of “ion-pairing” extraction, or two of them with “ion exchange” extraction may be the explanation of the mechanism of extraction with DES [33]. The “ion-pairing” mechanism of DES extraction of metal ions from the liquid phase is, for example, depicted by the following equation:

2 Me^2+^_(A)_ + 2 [Cl]^−^_(A)_ + 2 [COO]^−^_(O)_ = MeCl_2(A)_ + Me[COO]_2(O)_ (7)

Thus, with DES, it is possible to obtain better extraction of metal ions with two possible mechanisms. However, the volume of the organic phase after the extraction was always much lower than the volume of the aqueous phase.

The use of new bi-functional ILs did not improve the metal ions extraction efficiency. The results are slightly worse than those obtained for DES 2. The best results were obtained with [N_10,10,1,1_][Sal], where the extraction efficiency for Al(III) was *E*_Al_ = 34.0 wt%, *D*_Al_ = 0.34, and *E*_Cu_ = 6.0 wt%, *D*_Cu_ = 0.06 for Cu(II) and *E*_Zn_ = 6.4 wt%, *D*_Zn_ = 0.06 for Zn(II). Unfortunately, the extraction efficiency for silver ions was very low, *E*_Ag_ = 0.4 wt% with the distribution ratio equal to zero. The organic phase of [N_10,10,1,1_][Sal] was stripped twice and the results for both phases are shown. This is obvious that the method described above for DES 2 is much better. However, the separation of copper and silver from the other metals was unsuccessful. The separation of copper, zinc, and aluminum after the precipitation of silver with NaCl has to be solved.

The extraction from the solid material in the presence of bi-functional IL and the possible interaction with acidic anion may be interpreted as follows [38]:

Cu + 2 [N_10,10,1,1_]^+^ [Sal]^−^ + H_2_O_2_ + 2H^+^ = Cu[Sal]_2_ + 2 [N_10,10,1,1_]^+^ + 2 H_2_O (8)

The use of [N_10,10,1,1_][D2EHPA] unexpectedly was again not effective in this extraction (see Table 10). The extraction of all metal ions was not successful.

Nevertheless, the extraction of metal ions from solid material without leaching is a less-cost evolution of the recycling process for WPCB materials. However, extraction from the post-leaching solutions and from the solid material after leaching can result in almost 80–100 wt% extraction of metal ions from WPCBs materials. Unfortunately, in most of the processes, the distribution ratio is too low for technological use.

## 3. Materials and Methods

### 3.1. Materials

Samples of WPCBs came from Elemental H2Tech waste management in Poland. The WPCB blend was cut into small pieces and then crushed in a hydraulic press (see Figure 1). The final particles were put to the trial of thermal pre-treatment at the temperature *T* = 1023 K for 7 h in a Resistance Chamber Furnace (IZO), 16.1 kW. The mass of the sample after the thermal pre-treatment changed from 1.025 kg to 0.691 kg, a difference of 32.5%. In this way, undesirable coarse particles including plastic and different organic substances were eliminated (see Figure 2). Next, the solid material was additionally under pulverization process to obtain as small particles as possible.

Due to the heterogeneity of the solid WPCBs sample (see Figure 1) and the related difficulty in taking a representative sample for analysis, the metal content in the starting material used for ILs extraction was calculated on the basis of quantitative analysis (mass balance) of the residue and the solution obtained after leaching with aqua regia of the solid WPCBs sample (1 kg) after thermal pre-treatment (as a sum of metal content in the residue and solution after leaching). The metal ions contained in the aqueous solution were determined by the ICP-MS method using the Agilent 7900 inductively coupled plasma mass spectrometer. For determination of metal content in the solid phase (pre-analyzed for elemental composition by the SEM/EDS method), the Milestone UltraWAVE digestion system combined with the PerkinElmer AAnalyst 800 atomic absorption spectrometer (FAAS technique) was used. Metals were determined by the FAAS method after microwave-assisted digestion of the sample in an UltraWAVE mineralizer using concentrated nitric acid as solvent (acid-insoluble parts of the sample were leached in aqua regia and then fused with K_2_S_2_O_7_ and Na_2_CO_3_ to ensure complete dissolution of the sample prior to quantitative analysis).

WPCB solid samples were leached in two different ways: (I) with {4M H_2_SO_4_ + 100 g/dm^3^ (NH_2_)_2_CS + 13 g/dm^3^ Fe_2_(SO_4_)_3_} and (II) in two leaching steps, II (first step), with 5M HNO_3_ at the temperature *T* = 353 K, and II (second step), with {4M H_2_SO_4_ + 100 g/dm^3^ (NH_2_)_2_CS + 13 g/dm^3^ Fe_2_(SO_4_)_3_}. The obtained materials are presented in Figure 3 and Figure 4.

The post-leaching liquid phase (leaching I and II) of pH = 1 was alkalized with solid NaOH to pH = 3. As a result, two different solid phases were obtained: grey one and white one, which were separated from the post-leaching liquid phases. Next, the analyses by the SEM/EDS method (solid phases) and the ICP-OES technique (liquid phases) were carried out. The Jeol JSM-6490 LV scanning electron microscope (SEM) equipped with an energy-dispersive X-ray spectrometer (EDS), and the Thermo IRIS Advantage inductively coupled plasma optical emission spectrometer were used.

### 3.2. Reagents and Chemicals

The ILs used in this work were obtained from different firms such as Alfa Aesar, IoLiTec, Sigma Aldrich, or Heavy Water, or were synthesized in our laboratory. The chemical structure, name, abbreviation of the name, molar mass and mass fraction purity of the ILs are listed in Table 5. The synthesis and ^1^H NMR and ^13^C NMR spectra of the new three bi-functional ILs are presented in the Supplementary Material (SM). The list of solvents and other chemicals used is presented in Table 6. All other reagents employed in this work were of analytical grade. The water used was deionized by a Millipore purification system.

The samples of ILs were dried for 72 h at *T* = 340 K under reduced pressure, *p* = 6 kPa to remove volatile impurities and trace amounts of water. The water content in IL was analyzed by the Karl-Fischer titration technique (Metrohm, 716 DMS Titrino). The mass fraction of water in a sample was less than 800 × 10^−6^ g with an uncertainty of *u*(w.c.) = 10 × 10^−6^ g. The uncertainty of the temperature measurements was ±0.1 K. All weighing involved in the experimental work was carried out using a Mettler Toledo AB 204-S balance, with an accuracy of ±1 × 10^−4^ g.

### 3.3. Synthesis of DESs

DESs were proposed as primary solvents for the extraction of metal ions from the solid phase. The IL used for the preparation of DESs was dried under reduced pressure (10 hPa) at the temperature *T* = 323K for 8 h. The following DES mixtures were used: DES 1 (choline chloride, [N_2OH,1,1,1_][Cl]:lactic acid, 1:2 [39]); DES 2 (choline chloride, [N_2OH,1,1,1_][Cl]:malonic acid, 1:1 [40]. The synthesis of these DESs was presented in our earlier work [41].

### 3.4. Extraction Procedure

In all extraction experiments, 1.5 g of the solid phase was added to 15 cm^3^ of the aqueous phase and 15 cm^3^ of the organic phase (in some cases with the addition of acids, H_2_O_2_, DDACl). The mixture was shaken on a magnetic stirrer for 30 min or 2 h. The organic phase consisted of ILs immiscible with water, DES, or Cyanex 272 extractant with different additives. The mixtures were placed into a 100 cm^3^ jacketed glass cell with coated magnetic stirring bars for the stirring procedure, 5000 rpm. The vessels were sealed to avoid evaporation losses or the unwanted appearance of moisture from the atmosphere. The jacketed vessel was connected to a thermostatic water bath (PolyScience temperature controller) to maintain a constant temperature. The organic phase was stripped at different concentrations of sulphuric acid and under different conditions (time, temperature). After phase separation, samples of approximately 0.1–0.3 cm^3^ were taken for analysis from the aqueous phase after extraction and after stripping with H_2_SO_4_ using disposable plastic syringes with coupled stainless steel needles. The concentration of metal ions in the aqueous phase and stripped organic phase was determined with the IRIS Advantage inductively coupled plasma optical emission spectrometer (ICP-OES). The Litmus bromothymol blue (or other indicator papers sensitive to pH 1 to 10) were used to measure the pH of the solution after extraction.

### 3.5. Extraction with ILs or Organophosphorous Based Acid from the Post-Leaching Solutions

The results of the extraction of metal ions from the aqueous phase presented in our previous work [33], showed high efficiency of metal ions extraction with [P_6,6,6,14_][SCN] and [P_6,6,6,14_][Cyanex272] ILs.

In this work, the extraction was performed by magnetically stirring the of mixture containing 4 cm^3^ of the liquid leachate phase of pH = 3–7, 5 cm^3^ IL and 1 cm^3^ H_2_O_2_ (30 wt%). The [P_6,6,6,14_][Cyanex272] was used with toluene as a solvent due to its high viscosity (6.4 g of IL + 2.07 g of toluene). The mixture was placed into a 100 cm^3^ jacketed glass cell with a coated magnetic stirring bar and was stirred for 30 min, 5000 rpm at the temperature *T* = 303 K. Both phases after the separation were analyzed for the metal ions concentration. The organic phase was stripped with 1.2 M H_2_SO_4_. The volume ratio of acid to organic phase was 1:2 (2.5 cm^3^:5.0 cm^3^).

The best results were obtained with [P_6,6,6,14_][Cyanex272]/toluene and with the mixture (Cyanex 272 + diethyl phosphite ester). The mixture contained 6 cm^3^ of the liquid leachate phase of pH = 3, 4 cm^3^ of Cyanex 272, 1 cm^3^ of ester, 2 cm^3^ of naphtha (kerosene) and 1 cm^3^ H_2_O_2_. (30 wt%). The mixture was placed into a 100 cm^3^ jacketed glass cell with a coated magnetic stirring bar and was stirred for 30 min, 5000 rpm at the temperature *T* = 303 K. After the separation, both phases were analyzed for the metal ions concentration. The organic phase was stripped with 1.2 M H_2_SO_4_. The volume ratio of acid to organic phase was 1:2 (3.5 cm^3^:7.0 cm^3^). Results are presented in Table 7. The efficiency of metal ion extraction in wt% was calculated taking into account the determined density of the post-leaching solutions at pH = 3:1.217 g/cm^3^ (I)1.229 g/cm^3^ (II/first step), 1.212 g/cm^3^ (II/second step).

### 3.6. ABS Method of Extraction from the Post-Leaching Solutions

The 10 cm^3^ of the liquid leachate phase I of pH = 3, or the liquid leachate phase II (first/second step) was placed in a thermostated vessel. Then, 2.200 g of sodium chloride NaCl was, added in two portions. Next, 8.0076 g of [P_8,8,8,8_][Br], or 8.0069 g of [P_4,4,4,14_][Cl] was added and 1.5 cm^3^ H_2_O_2_ (30 wt%). The mixture was stirred for 2 h at the temperature *T* = 303 K. During the addition of NaCl, the IL separated as the upper layer of pH = 7. The extraction was carried out for 2 h at the temperature *T* = 318 K. The aqueous phase (approx. 10–11 cm^3^) was analyzed for the content of metal ions. The metal content in the upper organic phase was calculated from the difference between the aqueous phase and the liquid leachate phase. The results are presented in Table 8.

### 3.7. Extraction with DESs from the Solid Material

The solvent extraction and stripping were carried out in a 100 cm^3^ thermostated vessel with a coated magnetic stirrer bar under the water reflux at the temperature *T* = 333 K for 2 h. To 1.5 g of the solid material after thermal pre-treatment, 15 cm^3^ of DES 1 or DES 2 was added, 8 cm^3^ of DDACl (50 wt%) surfactant, 4.0 cm^3^ H_2_O_2_ (30 wt%), 3 cm^3^ of water and 2 M H_2_SO_4_ to pH = 3 or 5. For the solid phase, obtained after thermal pre-treatment at the temperature *T* = 1023 K for 7 h, the 0.4 g of Na_2_SO_4_ was added to DES 2 to get a higher transfer of metals from the solid to the liquid phase. The ratio (O:A = 1:1 *v*/*v*). The liquid phases after the separation were analyzed for the metal ions content. The results obtained at different modifications of extraction conditions are shown in Table 9 and Table 10.

### 3.8. Extraction with bi-Functional Ionic Liquids from the Solid Material

The solvent extraction from the solid material after thermal pre-treatment and stripping was carried out in a 100 cm^3^ thermostatic vessel with coated magnetic stirrer bar under the water reflux at the temperature *T* = 333 K for 2 h at pH = 6.

[N_10,10,1,1_][Sal] - to 1.5 g of the solid material 26 cm^3^ of viscous mixture of the IL (41.64 g) and toluene (30.38 g), 12.2 cm^3^ H_2_O, 6.9 cm^3^ of DDACl (50 wt%) surfactant, 6.9 cm^3^ H_2_O_2_ (30 wt%), were added. The ratio (O:A = 1:1). The liquid organic phase (30 cm^3^) was stripped with 15 cm^3^ 2M H_2_SO_4_ (O:Acid = 2:1 *v*/*v*) at the temperature *T* = 323 K for 20 min. The first aqueous phase (18 cm^3^) and the new acidic aqueous phase (30 cm^3^) were analyzed for the metal ions concentration. The results are shown in Table 9 and Table 10.

[N_10,10,1,1_][D2EHPA]- to 1.5 g of the solid material 15 cm^3^ of the IL and 1 cm^3^ of toluene, 8 cm^3^ H_2_O, 4 cm^3^ of DDACl (50 wt%) surfactant, 4 cm^3^ H_2_O_2_ (30 wt%), were added. The ratio (O:A = 1:1 *v*/*v*). Liquid organic phase (15 cm^3^) was stripped with 7.5 cm^3^ 2M H_2_SO_4_ (O:Acid = 2:1) at the temperature *T* = 323 K for 20 min. The first aqueous phase (18 cm^3^) and the new acidic aqueous phase (30 cm^3^) were analyzed for the metal ions concentration. The results are shown in Table 9 and Table 10.

[N_10,10,1,1_][Cyanex272] - to 1.5 g of the solid material 15 cm^3^ of the IL and 1 cm^3^ of toluene, 8 cm^3^ H_2_O, 4 cm^3^ of DDACl (50 wt%) surfactant, 4 cm^3^ H_2_O_2_ (30 wt%), were added. The ratio (O:A = 1:1). The liquid organic phase (16 cm^3^) was stripped with 8 cm^3^ 2 M H_2_SO_4_ (O:Acid = 2:1 *v*/*v*) at the temperature *T* = 323 K for 20 min. The results are shown in Table 9 and Table 10.

The extraction efficiency was calculated from the sum of the metal content in the aqueous phase and the stripped organic phase in relation to the metal content in the solid phase sample in the starting material, obtained after thermal pre-treatment at the temperature *T* = 1023 K for 7 h.

## 4. Conclusions and Future Perspectives

In this study, we used ionic liquids (IL), the ABS method with NaCl, to leach copper, silver and other metals from WPCBs after thermal pre-treatment (*T* = 1023 K, 7 h) and after leaching I using {4M H_2_SO_4_ +100 g/dm^3^ (NH_2_)_2_CS + 13 g/dm^3^ Fe_2_(SO_4_)_3_}, or leaching II (first step) and II (second step) with 5M HNO_3_ and {4M H_2_SO_4_ +100 g/dm^3^ (NH_2_)_2_CS +13 g/dm^3^ Fe_2_(SO_4_)_3_}, as well as the DESs and bi-functional ILs for the extraction of metal ions from the solid material after thermal pre-treatment and leaching, or without leaching. ILs known for their high extraction efficiency of metal ions were selected, [P_6,6,6,14_][SCN], Aliquat 336, [P_6,6,6,14_][Cyanex272], Cyanex 272, and two [P_4,4,4,14_][Cl], [P_8,8,8,8_][Br] in the ABS method. The extraction efficiency of Cu(II), Ag(I), Al(III), Zn(II) from the liquid leachate phase with [P_6,6,6,14_][Cyanex272]/toluene was higher than 60 wt% at the temperature *T* = 303 K for 30 min at pH = 7. The extraction of Ag(I) from the liquid leachate phase with a mixture (Cyanex 272 + diethyl phosphite ester) was *E*_Ag_ = 100 wt%, with distribution ratio *D* = 1 at the temperature *T* = 303 K for 30 min at pH = 3. The ABS method of salting extraction of metal ions from the liquid leachate phases was 80–100 wt% for all metals including 96 wt%–100 wt% for Cu(II) and Ag(I) at the temperature *T* = 303 K, for 30 min at pH = 3.

The extraction from the solid material after thermal pre-treatment was presented with two DESs, DES 1 (choline chloride + lactic acid) and DES 2 (choline chloride + malonic acid). Better metal extraction results were obtained with DES 2 at pH = 5 at the temperature *T* = 333 K. The efficiency of extraction of various metals from the solid material after thermal pre-treatment and I leaching was: *E*_Cu_ = 15.8 wt%, *E*_Ag_ = 20.1 wt%, *E*_Al_ = 48.9 wt%, *E*_Fe_ = 24.7 wt%. Factors influencing the metal extraction efficiency were investigated in detail, and the addition of H_2_O_2_ and DDACL to the extraction effects was also examined. The results showed that the extraction with DES 2 from the solid material without leaching is slightly worse than those after leaching: for Al(III), *E*_Al_ = 67.3 wt%, *E*_Cu_ = 9.6 wt% and *E*_Ag_ = 14.2 wt% at pH = 5. Only one bi-functional IL, [N_10,10,1,1_][Sal] could successfully leach metals out from the solid material only after thermal pre-treatment, where the extraction efficiency for Al(III) was *E*_Al_ = 34.0 wt%, for Cu(II) *E*_Cu_ = 6.0 wt%, for Zn(II) *E*_Zn_ = 6.4 wt% and for Ag(I) *E*_Ag_ = 0.4 wt%. The extraction efficiency (*E*) and distribution ratio (*D*) of many Cu(II), Ag(I), Al(III) and Zn(II) extraction processes were examined. The [P_6,6,6,14_][SCN] and Aliquat 336, ILs proposed for this extraction was not effective.

The WPCBs particle size and the leaching solvent had a similar effect on the metals leaching performance, while the amount of DDACl addition showed no influence. This innovation provides a new option for recovering valuable metals such as copper or silver from WPCBs materials. The processes presented in this work may be used in a new technology of the recovery of metal ions from WPCBs in the form of a temperature pre-treated solid material. The leaching process is time-consuming, very costly and toxic. The absence of acid leaching results in a lower metal extraction efficiency. All these results show that the solid phase extraction after leaching plus leachate extraction can yield 90–100% metal extraction, or only about 10–15% major metals such as Cu and Ag with solid material extraction without leaching. These results ensure a feasible and future-proof industrial application.

## Figures and Tables

**Table 1:** Metal content in the starting WPCBs material after thermal pre-treatment at the temperature *T* = 1023 K, 7 h and leaching I (4M H_2_SO_4_ + 100 g/dm^3^ (NH_2_)_2_CS + 13 g/dm^3^ Fe_2_(SO_4_)_3_), and after two steps of leaching II (5M HNO_3_ and (4M H_2_SO_4_ + 100 g/dm^3^ (NH_2_)_2_CS + 13 g/dm^3^ Fe_2_(SO_4_)_3_)), respectively) (microwave digestion/FAAS and ICP-MS methods).

Metal Content							Mass of Sample
Al	Fe	Ni	Cu	Zn	Ag	Pb	kg
g/kg					mg/kg	g/kg	
Starting material							
81.9	79.4	1.47	224	18.1	483	7.8	1.000
Solid material after thermal pre-treatment							
122	119	2.20	335	27.0	721	11.6	0.670
Solid material after the leaching I							
130	106	1.69	235	20.6	766	12.7	0.575
Solid material after the leaching II (second step)							
96.9	11.5	< *	<	<	<	<	0.328

* The symbol ‘‘<” indicates the element content below the limit of quantification of the test method used.

**Table 2:** Metal content in the liquid phases after leaching processes (ICP-MS method).

Metal Content									
mg/kg									
Al	Fe	Ni	Cu	Zn	Pd	Ag	Sn	Au	Pb
After the I leaching									
864	5059	59.2	10,505	733	0.57	5.0	533	3.3	53.0
After the II leaching (first step)									
6110	6985	213	29,544	2205	<0.5	0.5	59.9	<1	1194
After the II leaching (second step)									
254	7008	51.4	904	257	1.25	100	834	2.7	14.8

**Table 3:** Content of elements in the solid phases after alkalization of the post-leaching solutions to pH = 3 (SEM/EDS method).

Element	I (Grey) wt%	Element	I (White) wt%	Element	II First Step (Brown) wt%	Element	II Second Step (Grey) wt%	Element	II Second Step (White) wt%
O	33–34	O	39–41	O	48–49	O	40	O	38–42
Na	22	Na	29–30	Na	13–17	Na	28–29	Na	23–30
S	35–36	S	29–31	Al	11–14	S	30–31	S	30–37
Fe	0.5	Fe	0.2–0.4	Si	3–8	Fe	0.5–1	Fe	0.5–2
Cu	7–8	Cu	0.3–0.4	Ca	< *	Cu	0.5–1	Cu	0.5
Sn	0.8–1			Fe	9–11				
				Cu	7–11				

* Content below the limit of quantification of the test method used.

**Table 4:** Metal content in the liquid phases after alkalization of the post-leaching solutions to pH = 3 (ICP-OES method.

**Metal content in the liquid leachate phase I**						
g/kg	g/kg	g/kg	g/kg	g/kg	mg/kg	g/kg
Al	Fe	Ni	Cu	Zn	Ag	Pb
0.808	5.556	0.0764	0.776	0.8055	0.7069	0.0402
**Metal content in the liquid leachate phase II (first step)**						
g/kg	g/kg	g/kg	g/kg	g/kg	mg/kg	g/kg
Al	Fe	Ni	Cu	Zn	Ag	Pb
3.206	3.596	-	21.146	1.806	0.1383	-
**Metal content in the liquid leachate phase II (second step)**						
g/kg	g/kg	g/kg	g/kg	g/kg	mg/kg	g/kg
Al	Fe	Ni	Cu	Zn	Ag	Pb
0.0965	9.7897	-	1.2833	0.4305	146.804	-

**Table 5:** Data on the ionic liquids used: structure, name, abbreviation of name, supplier, CAS number, molar mass (M), mass fraction purity (as stated by the supplier).

Chemical Structure	Name, Abbreviation, Supplier, CAS Number	M/(g mol^−1^)	Purity in Mass Percent (%)
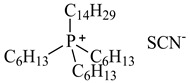	Trihexyltetradecylphosphonium thiocyanate, [P_6,6,6,14_][SCN], synthesized [9]	542.06	>95
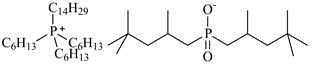	Trihexyltetradecylphosphonium bis(2,4,4-trimethylpentyl)phosphinate, [P_6,6,6,14_][Cyanex272], ([P_6,6,6,14_][BTMPP]), IoLiTec, CAS: 465527-59-7	773.27	>90
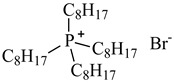	Tetraoctylphosphonium bromide, [P_8,8,8,8_][Br], IoLiTec, CAS: 23906-97-0	563.76	>95
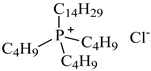	Tributyltetradecylphosphonium chloride, [P_4,4,4,14_][Cl], IoLiTec, CAS: 81741-28-8	435.24	>95
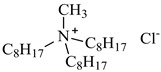	Methyltrioctylammonium chloride, Aliquat 336, [N_1,8,8,8_][Cl], Alfa Aesar, CAS: 63393-96-4	404.17	>97
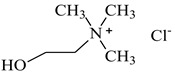	Choline chloride, [N_2OH,1,1,1_][Cl], Sigma-Aldrich, CAS: 67-48-1	139.62	>98
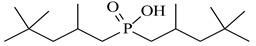	Bis(2,4,4-trimethylpentyl)phosphinic acid, Cyanex 272, Chem Scene LLC, CAS: 83411-71-6	290.42	90
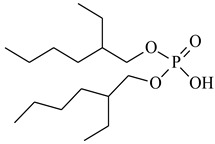	Bis(2-ethylhexyl) phosphate, D2EHPA, Heavy Water, CAS: 298-07-7	322.40	>95
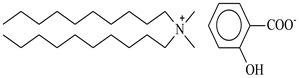	Didecyldimethylammonium salicylate, [N_10,10,1,1_][Sal], C_29_H_53_NO_3_, synthesized	463.83	>95
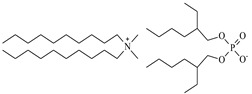	Didecyldimethylammonium bis(2-ethylhexyl) phosphate, [N_10,10,1,1_][DEHPA], C_38_H_82_NO_4_P, synthesized	648.13	>95
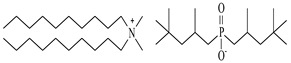	Didecyldimethylammonium bis(2,4,4-trimethylpentyl)phosphinate, [N_10,10,1,1_][Cyanex272], synthesized	616.12	>95

**Table 6:** Data on the chemicals used: name, supplier, CAS number, molecular mass (M), mass fraction purity (as stated by the supplier).

Name, Supplier, CAS Number	M/(g mol^−1^)	Purity in Mass Percent (%)
Lactic acid, C_2_H_4_OHCOOH, Sigma-Aldrich, *CAS 50-21-5*	90.08	purified, 333 K, 13 hPa, 85 wt% aq. solution
Malonic acid, C_3_H_4_O_4_, Reachim, CAS 141-82-2	104.06	99.0
Diethyl phosphite, C_4_H_11_PO_3_ Sigma-Aldrich, CAS: 762-04-9	138.10	98.0
Didecyldimethylammonium chloride, [N_10,10,1,1_][Cl], DDACl, Alpinus Sp. z o.o., CAS 7173-51-5	362.16	50 wt% aq. solution
Hydrogen peroxide, H_2_O_2_, Chempur, CAS 7722-84-1	34.01	30 wt% aq. solution
Sulfuric acid, H_2_SO_4_, Riedel-de Haën, CAS 7664-93-9	98.08	96.0
Sodium sulfate, Na_2_SO_4_, Chempur, CAS 7757-82-6	142.04	99.0
Sodium hydroxide, NaOH, POCh, CAS 1310-73-2	40.0	98.8
Toluene, C_6_H_5_CH_3_, Chempur, CAS 108-88-3	92.14	98.8

**Table 7:** Results of metals extraction with ILs from the post-leaching solution I, extraction efficiency (*E*/wt%), distribution ratio (*D*), pH of the aqueous phase after extraction at the temperature *T* = 303 K.

IL	Ion	m_0_/mg	m_E,O_/mg	*E*/wt%	*D*	pH
[P_6,6,6,14_][Cyanex272]/toluene	Cu(II)	3.78	2.24	59.3	0.59	7
	Ag(I)	0.003	0.002	66.7	0.67	
	Al(III)	5.93	5.37	90.5	0.91	
	Fe(II)	27.04	4.67	17.3	0.17	
	Zn(II)	4.92	4.72	95.9	0.96	
Cyanex 272 + ester	Cu(II)	5.66	0.55	9.7	0.01	3
	Ag(I)	0.005	0.005	100.0	1.00	
	Al(III)	5.90	0.72	12.2	0.12	
	Fe(II)	40.56	4.79	11.8	0.12	
	Zn(II)	5.88	3.04	51.7	0.52	

**Table 8:** Results of metals extraction with ABS method from the post-leaching solutions I and II at pH = 3, extraction efficiency (*E*/wt%), pH of the aqueous phase after extraction at the temperature *T* = 303 K.

Mixture	Ion	m_0_/mg	m_E,A_/mg	m_E,O_/mg	*E*/wt%	pH
Metal content in the liquid leachate phase I						
{[P_8,8,8,8_][Br] + NaCl + H_2_O_2_ + leachate I}	Cu(II)	9.44	0.08	9.36	99.1	3
	Ag(I)	0.009	<0.0004	>0.008	88.9	
	Al(III)	14.00	13.86	0	99.0 *	
	Fe(II)	67.60	4.58	63.02	93.2	
	Zn(II)	9.80	0.011	9.79	99.9	
{[P_4,4,4,14_][Cl] + NaCl + H_2_O_2_ + leachate I}	Cu(II)	9.44	0.003	9.44	100.0	3
	Ag(I)	0.009	<0.0004	>0.008	93.3	
	Al(III)	13.02	12.98	0	99.7 *	
	Fe(II)	67.60	3.96	63.64	94.1	
	Zn(II)	9.80	0.011	9.79	99.9	
Metal content in the liquid leachate phase II (first step)						
{[P_8,8,8,8_][Br] + NaCl + H_2_O_2_ + leachate II (first step)}	Cu(II)	259.90	1.00	258.90 **	99.6	3
	Ag(I)	0.0017	0.0001	0.0016 **	94.1	
	Al(III)	39.40	5.17	34.23 **	86.9	
	Fe(II)	44.20	2.85	41.35 **	93.5	
	Zn(II)	22.20	0.0115	22.19 **	99.9	
{[P_8,8,8,8_][Br] + NaCl + leachate II (first step)}	Cu(II)	259.90	77.20	182.7 **	70.3	3
	Ag(I)	0.0017	0.001	0.0007 **	41.2	
	Al(III)	39.40	18.25	21.15 **	39.4	
	Fe(II)	44.20	17.38	26.82 **	60.7	
	Zn(II)	22.20	12.30	9.90 **	44.6	
{[P_4,4,4,14_][Cl] + NaCl + H_2_O_2_ + leachate II (first step)}	Cu(II)	259.90	9.24	250.66	96.4	3
	Ag(I)	0.002	0	0.002	100.0	
	Al(III)	39.40	30.80	8.60	78.2 *	
	Fe(II)	44.20	24.09	20.11	45.5	
	Zn(II)	22.20	0.10	22.10	99.5	
Metal content in liquid leachate phase II (second step)						
{[P_4,4,4,14_][Cl] + NaCl + H_2_O_2_ + leachate II (second step)}	Cu(II)	15.56	0.30	15.26	98.1	3
	Ag(I)	1.78	0.0004	1.78	100.0	
	Al(III)	1.17	1.04	0.132	88.9 *	
	Fe(II)	118.7	8.02	110.7	93.3	
	Zn(II)	5.22	0.003	5.22	100.0	
{[P_8,8,8,8_][Br] + NaCl + H_2_O_2_ + leachate II (second step)}	Cu(II)	15.56	0.46	15.10	97.0	3
	Ag(I)	1.78	0.0025	1.78	100.0	
	Al(III)	1.85	1.80	0	97.3 *	
	Fe(II)	118.7	20.70	98.00	82.6	
	Zn(II)	5.22	0.004	5.22	100.0	

* Calculated from the aqueous phase. ** Brown thick solution.

**Table 9:** Results of metals extraction from the solid WPCBs material after thermal pre-treatment (*T* = 1023 K, 7 h) and the I leaching {4M H_2_SO_4_ + 100 g/dm^3^ (NH_2_)_2_CS + 13 g/dm^3^ Fe_2_(SO_4_)_3_}, with DESs and bi-functional ILs, extraction efficiency (*E*/wt%), distribution ratio (*D*) and pH of the aqueous phase after extraction.

Mixture	Ion	m_0_/mg	m_E,O_/mg	*E*/wt%	*D*	pH
DES 1	Cu(II)	352.5	15.07	4.3	0.04	2.5
	Ag(I)	1.149	0.06	5.2	0.05	
	Al(III)	195.0	99.94	51.2	0.51	
	Fe(II)	159.0	44.01	27.7	0.28	
	Zn(II)	30.90	1.092	3.5	0.03	
DES 2	Cu(II)	352.5	31.08	8.8	0.09	2.5
	Ag(I)	1.149	0.303	26.4	0.26	
	Al(III)	195.0	178.4	91.5	0.91	
	Fe(II)	159.0	31.29	19.7	0.20	
	Zn(II)	30.90	0.72	2.3	0.02	
DES 1	Cu(II)	352.5	12.31	3.5	0.03	5
	Ag(I)	1.149	0.15	13.0	0.13	
	Al(III)	195.0	102.8	52.7	0.53	
	Fe(II)	159.0	38.19	24.0	0.24	
	Zn(II)	30.90	1.05	3.40	0.03	
DES 2	Cu(II)	352.5	55.66	15.8	0.16	5
	Ag(I)	1.149	0.231	20.1	0.20	
	Al(III)	195.0	95.32	48.9	0.49	
	Fe(II)	159.0	39.36	24.7	0.25	
	Zn(II)	30.90	0.623	2.0	0.02	
[N_10,10,1,1_][Sal]	Cu(II)	352.5	32.37	9.2	0.09	6
	Ag(I)	1.149	0.0022	0.2	0	
	Al(III)	195.0	49.68	25.5	0.25	
	Fe(II)	159.0	12.43	7.8	0.08	
	Zn(II)	30.90	0.50	1.6	0.02	
[N_10,10,1,1_][D2EHPA]	Cu(II)	352.5	8.96	2.5	0.02	6
	Ag(I)	1.149	0.00056	0.05	0	
	Al(III)	195.0	7.78	4.0	0.04	
	Fe(II)	159.0	1.64	1.0	0.01	
	Zn(II)	30.90	0.133	0.4	0	
[N_10,10,1,1_][Cyanex272]	Cu(II)	352.5	13.33	3.8	0.04	6
	Ag(I)	1.149	0	0	0	
	Al(III)	195.0	16.77	8.6	0.09	
	Fe(II)	159.0	2.34	1.5	0.01	
	Zn(II)	30.90	0.19	0.6	0	

**Table 10:** Results of metals extraction from the solid WPCBs material after thermal pre-treatment (*T* = 1023 K, 7 h), with DES 2 and bi-functional ILs, extraction efficiency (*E*/wt%), distribution ratio (*D*) and pH of the aqueous phase after extraction.

Mixture	Ion	m_0_/mg	m_E,O_/mg	*E*/wt%	*D*	pH
DES 2	Cu(II)	502.50	48.45	9.6	0.09	5
	Ag(I)	1.082	0.154	14.2	0.14	
	Al(III)	183.00	123.13	67.3	0.67	
	Fe(II)	178.50	18.06	10.1	0.10	
	Zn(II)	40.50	2.63	6.5	0.06	
DES 2 + Na_2_SO_4_	Cu(II)	502.50	27.35	5.4	0.05	5
	Ag(I)	1.082	0.115	10.6	0.11	
	Al(III)	183.00	86.33	47.2	0.47	
	Fe(II)	178.50	12.81	7.2	0.07	
	Zn(II)	40.50	2.15	5.3	0.05	
[N_10,10,1,1_][Sal]	Cu(II)	502.50	30.16	6.0	0.06	6
	Ag(I)	1.082	0.0044	0.4	0	
	Al(III)	183.00	62.30	34.0	0.34	
	Fe(II)	178.50	6.65	3.7	0.03	
	Zn(II)	40.50	2.61	6.4	0.06	
[N_10,10,1,1_][D2EHPA]	Cu(II)	502.50	5.77	1.1	0.01	6
	Ag(I)	1.082	0.0007	0.06	0	
	Al(III)	183.00	18.48	10.1	0.10	
	Fe(II)	178.50	1.37	0.8	0.01	
	Zn(II)	40.50	0.97	2.4	0.02	
[N_10,10,1,1_][Cyanex272]	Cu(II)	502.50	35.30	7.0	0.07	6
	Ag(I)	1.082	0.0015	0.1	0	
	Al(III)	183.00	33.86	18.5	0.18	
	Fe(II)	178.50	1.40	0.8	0.01	
	Zn(II)	40.50	0.79	1.9	0.02	

## Data Availability

Data is contained within the article or supplementary material.

## Supplementary Materials

The following supporting information can be downloaded at: https://www.mdpi.com/article/10.3390/molecules27154984/s1—The synthesis of three bi-functional ILs with NMR is presented.
