# Supplementary material for: Recovery of Metals from Electronic Waste-Printed Circuit Boards by Ionic Liquids, DESs and Organophosphorous-Based Acid Extraction
Source: Molecules. 2022 Aug 5;27(15):4984. doi: 10.3390/molecules27154984 (PMC9370793; doi:10.3390/molecules27154984)

Special edition of ***Molecules*** entitled "Application of Ionic Liquid for Extraction and Separation."

Supplementary Material

Recovery of metals from electronic waste - printed circuit boards by ionic liquids, DESs and organophosphorous-based acid extraction

Aneta Łukomska[1], Anna Wiśniewska[1], Zbigniew Dąbrowski[1], Jakub Lach[1], Kamil Wróbel[1], Dorota Kolasa[1], Urszula Domańska[1,*]

[1]ŁUKASIEWICZ Research Network – Industrial Chemistry Institute, Rydygiera 8, 01-793 Warsaw, Poland.

S1. SYNTHESIS OF NEW BI-FUNCTIONAL IONIC LIQUIDS

S1.1. The synthesis of didecyldimethylammonium salicylate ($[N_{10,10,1,1}][Sal]$, $C_{29}H_{53}NO_3$)

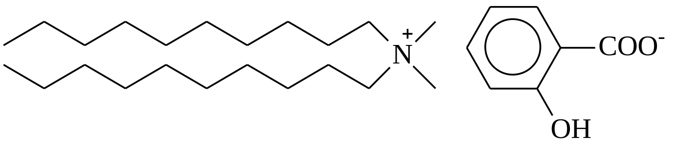

M = 463.83

Salicylic acid (13.81 g, 0.1 mol) and water (65 cm^3) were placed in a 500 cm^3 round bottom flask equipped with a magnetic stirrer, cooler condenser and thermometer. To the obtained suspension 5.61 g (0.1 mol) of potassium hydroxide dissolved in water (30 cm^3) was added. The mixture was stirred at T = 343 K for 2 h until salicylic acid was completely dissolved /until the complete dissolution of the salicylic acid. Next, 72.44 g of a 50 % aqueous solution of didecyldimethylammonium chloride (36.22 g, 0.1 mol of $[N_{10,10,1,1}][Cl]$) was added. There was immediate cloudiness upon the addition of the didecyldimethylammonium chloride solution. The mixture was stirred at T = 333 K for 6 h, then cooled to T = 298 K and 100 cm^3 of dichloromethane was added. Two phases were separated. The organic layer containing the desired product was washed 2 times (2 × 50 cm^3) with water to remove the potassium chloride. The solvent was removed by distillation under reduced pressure. The residue was dried at 1.3 hPa (333 K, 10 h). 43.40 g (0.094 mol) of didecyldimethylammonium salicylate was obtained in the solid phase. Yield 94.0 %.

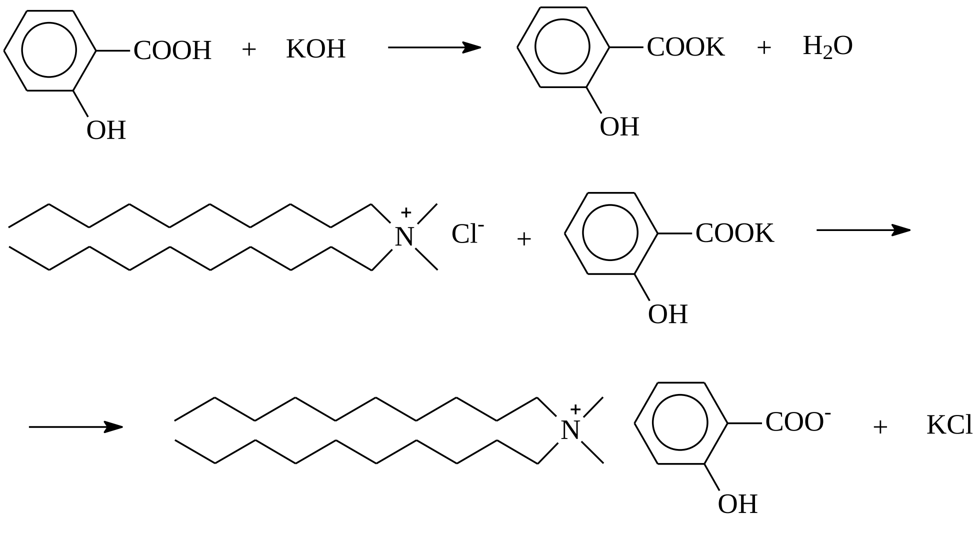

^{1}H NMR ($CDCl_3$, 500 MHz), δ: 0.83 (t, 6H, 2 × CH_3, J = 5.0 Hz), 1.12-1.27 (m, 28H, 14 × CH_2), 1.48-1.56 (m, 4H, 2 × CH_2), 3.11 (s, 6H, 2 × $\mathbf{CH_3}[N^+]$), 3.18-3.21 (m, 4H, 2 × $\mathbf{CH_2}[N^+]$), 6.69 (dt, 1H, Ar, J_1

= 7.5 Hz, J_2 = 1.0 Hz), 6.77 (dd, 1H, Ar, J_1 = 10.0 Hz, J_2 = 2.5 Hz), 7.20 (dt, 1H, Ar, J_1 = 7.5 Hz, J_2 = 1.0 Hz), 7.85 (dd, 1H, Ar, J_1 = 10.0 Hz, J_2 = 2.5 Hz), 10.49 (s, 1H, ArO**H**) ppm.

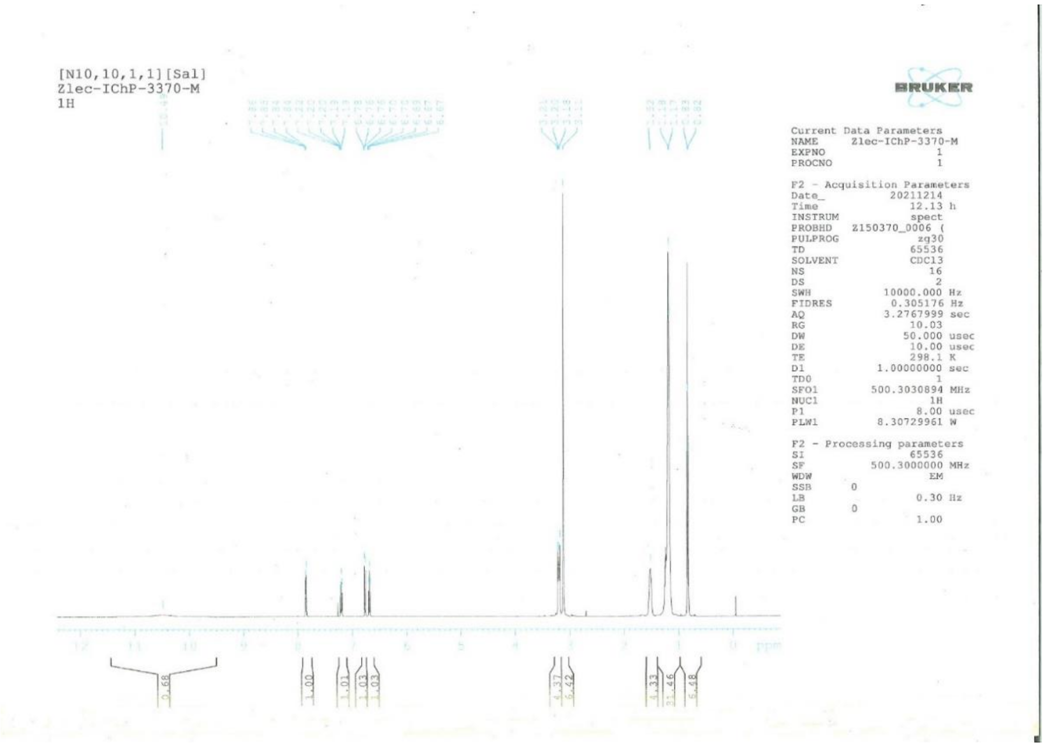

^{13}C NMR ($CDCl_3$, 125.8 MHz), δ: 14.06 (2 × CH_3), 22.60 (2 × CH_2), 22.62 (2 × CH_2), 26.12 (2 × CH_2), 29.06 (2 × CH_2), 29.18 (2 × CH_2), 29.29 (2 × CH_2), 29.34 (2 × CH_2), 31.78 (2 × CH_2), 50.91 (2 × **CH_3**[N^+]), 63.92 (2 × **CH_2**[N^+]), 116.41 (CH, Ar), 117.55 (CH, Ar), 118.69 (C, Ar), 130.54 (CH, Ar), 132.75 (CH, Ar), 162.13 (C, Ar-OH), 173.65 ([**C=O**]O-) ppm.

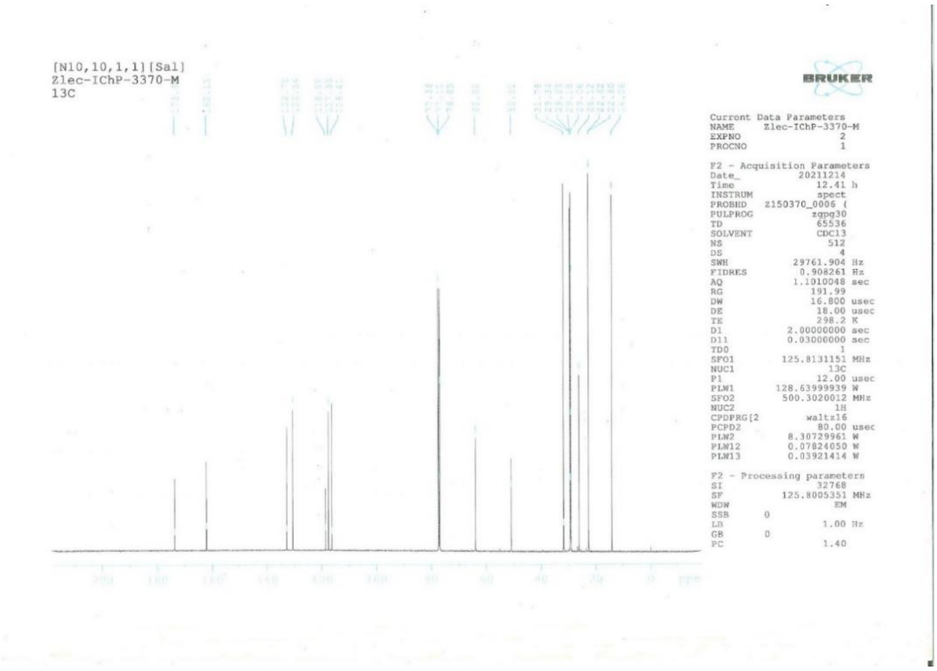

S1.2. The synthesis of didecyldimethylammonium bis(2-ethylhexyl)phosphate ([$N_{10,10,1,1}$][D2EHPA], $C_{38}H_{82}NO_4P$)

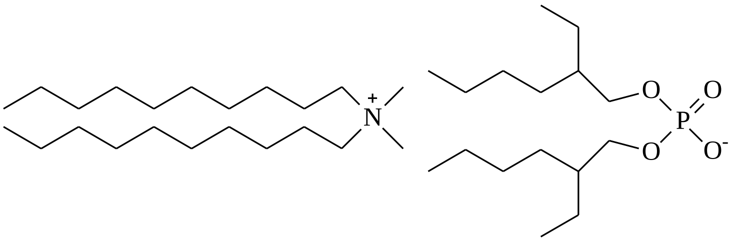

M = 648.13

32.24 g (0.1 mol) of bis(2-ethylhexyl)phosphoric acid were placed in a 500 cm^3 round bottom flask equipped with a magnetic stirrer, cooler condenser and thermometer. Next, the solution of potassium hydroxide (5.61 g, 0.1 mol) in water (50 cm^3) was added. The thick and glassy mixture was stirred magnetically at T = 343 K for 2 h. Next, 72.44 g of a 50% aqueous solution of didecyldimethylammonium chloride (36.22 g, 0.1 mol of [$N_{10,10,1,1}$][Cl]) was added. The mixture was stirred at T = 338 K for 6 h, then cooled to T = 298 K and 100 cm^3 of dichloromethane were added. Two phases were separated. The organic layer containing the desired product was washed 2 times (2 × 50 cm^3) with water to remove the potassium chloride. The solvent was removed by distillation under reduced pressure. The residue was dried at 1.3 hPa (333 K, 10 h). 62.39 g (0.096 mol) of didecyldimethylammonium bis(2-ethylhexyl)phosphate was obtained in the form of a clear thick liquid. Yield 96.0%.

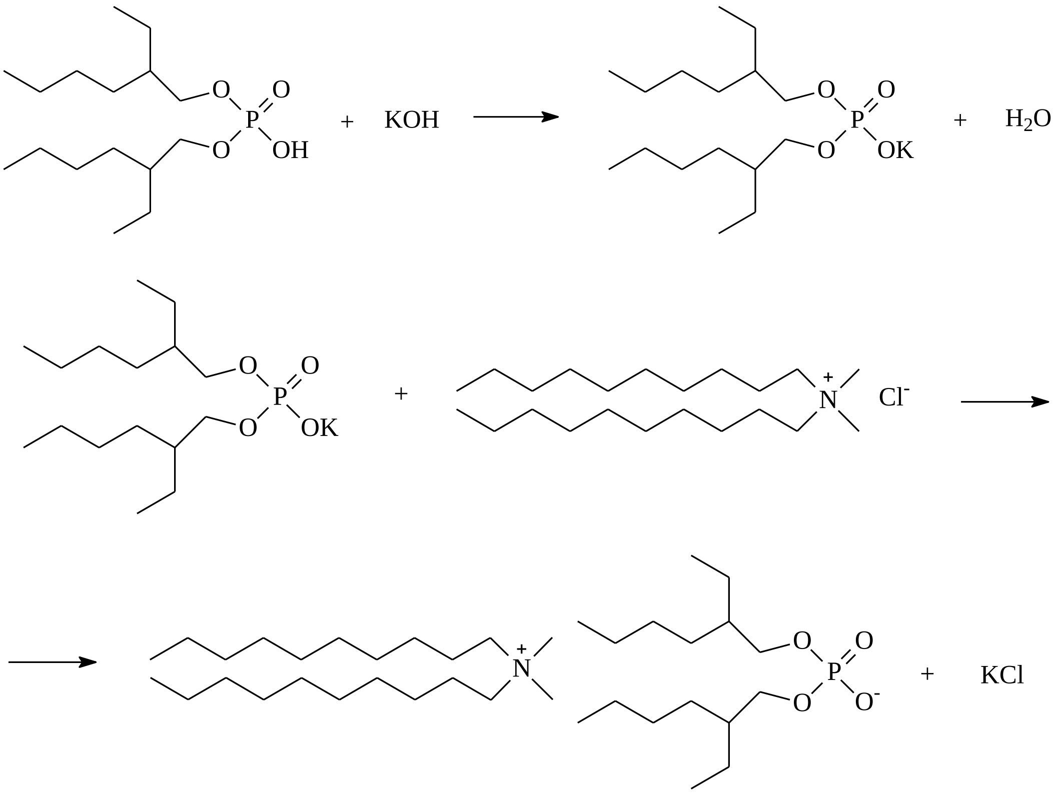

^{1}H NMR ($CDCl_3$, 500 MHz), δ: 0.77-0.81 (m, 18H, 6 × CH_3), 1.13-1.28 (m, 42H), 1.33-1.39 (m, 2H), 1.41-1.46 (m, 2H), 1.56-1.62 (m, 4H, 2 × CH_2), 3.26 (s, 6H, 2 × **CH_3**[N^+]), 3.32-3.35 (m, 4H, 2 × **CH_2**[N^+]), 3.61-3.69 (m, 4H, 2 × **CH_2**O) ppm.

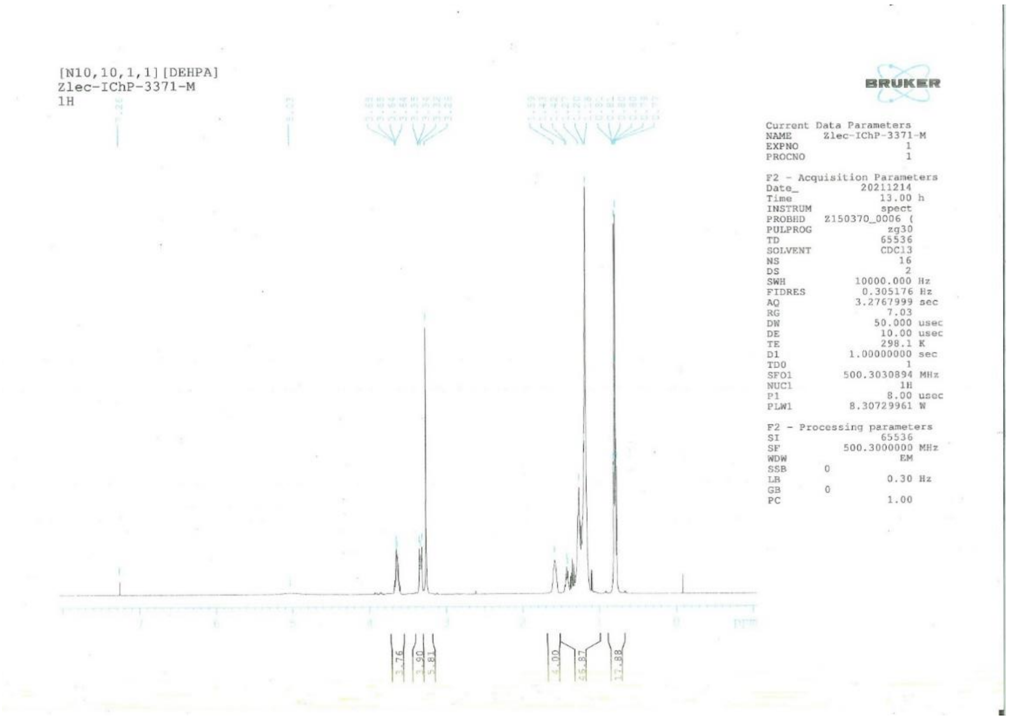

^{13}C NMR ($CDCl_3$, 125.8 MHz), δ: 10.83 (2 × CH_3), 13.99 (2 × CH_3), 14.05 (2 × CH_3), 22.56 (2 × CH_2), 22.68 (2 × CH_2), 23.08 (2 × CH_2), 23.26 (2 × CH_2), 26.25 (2 × CH_2), 28.99 (2 × CH_2), 29.17 (2 × CH_2), 29.20 (2 × CH_2), 29.34 (2 × CH_2), 29.37 (2 × CH_2), 30.04 (2 × CH_2), 31.75 (2 × CH_2), 40.29 (CH), 40.35 (CH), 51.14 (2 × **CH_3**[N^+]), 63.21 (2 × **CH_2**[N^+]), 67.55 (CH_2O), 67.55 (CH_2O) ppm

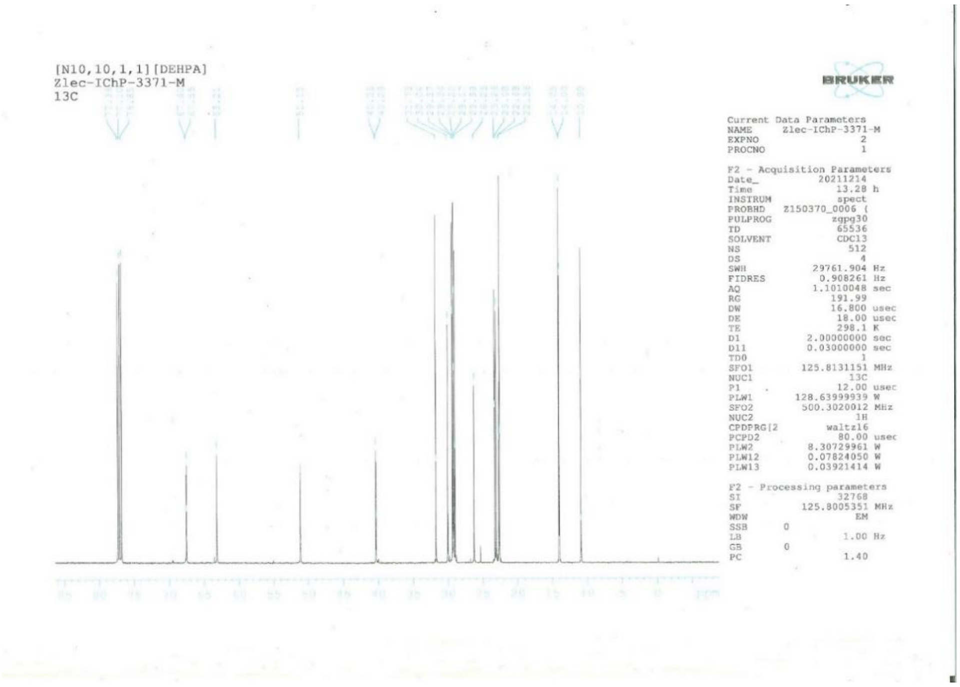

S1.3. The synthesis of didecyldimethylammonium bis(2-2,4,4-trimethylpentyl)phosphinate ([$N_{10,10,1,1}$][Cyanex272], $C_{38}H_{82}NO_2P$)

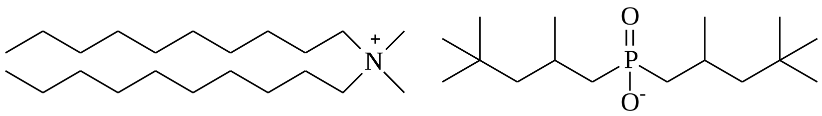

M = 616.12

16.13 g (0.05 mol) of bis(2,4,4-trimethylopentyl)phosphinic acid (Cyanex 272, 90%) and a solution of potassium hydroxide (2.80g, 0.05 mol) in water (30 cm^3) was placed in a 250 cm^3 round bottom flask equipped with a magnetic stirrer, cooler condenser, and thermometer The mixture was stirred at T = 338 K for 3 h. Next, 36.22 g of a 50% aqueous solution of didecyldimethylammonium chloride (18.11 g, 0.05 mol of [$N_{10,10,1,1}$][Cl]) was added. The cloudiness occurred after the addition of the ammonium chloride solution. The mixture was stirred at T = 338 K for 6 h. After cooling the mixture, dichlorometane (60 cm^3) was added and 2 phases were separated. The organic phase containing the ionic liquid was washed 2 times with water (2 × 30 cm^3). The solvent was removed by distillation under pressure and the residue was dried at 1.3 hPa for 10 h (333 K). 31.68 g (0.0495 mol) of didecyldimethylammonium bis(2-2,4,4-trimethylpentyl)phosphinate were obtained in the form of a clear, thick liquid. Yield 99%.

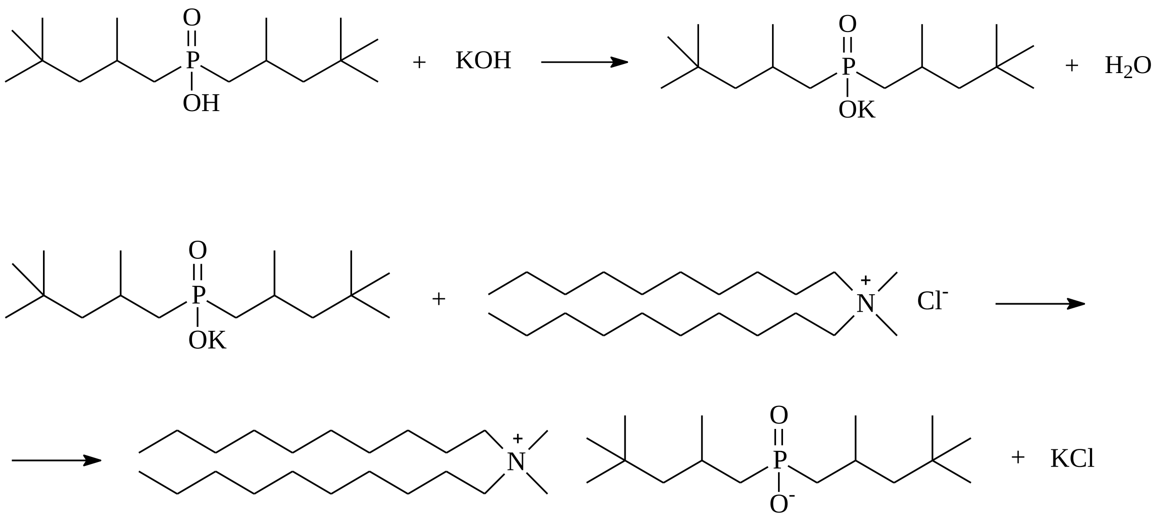

^{1}H NMR ($CDCl_3$, 500 MHz), δ: 0.77-0.84 (m, 24 H, 8 × CH_3), 1.00-1.04 (m, 8H), 1.13-1.30 (m, 32H, 16 × CH_2), 1.45-1.52 (m, 2H), 1.54-1.60 (m, 4H), 1.88-1.93 (m, 2H), 3.28 (s, 6H, 2 × **CH_3**[N^+]), 3.34-3.37 m, 4H, 2 × **CH_2**[N^+]) ppm.

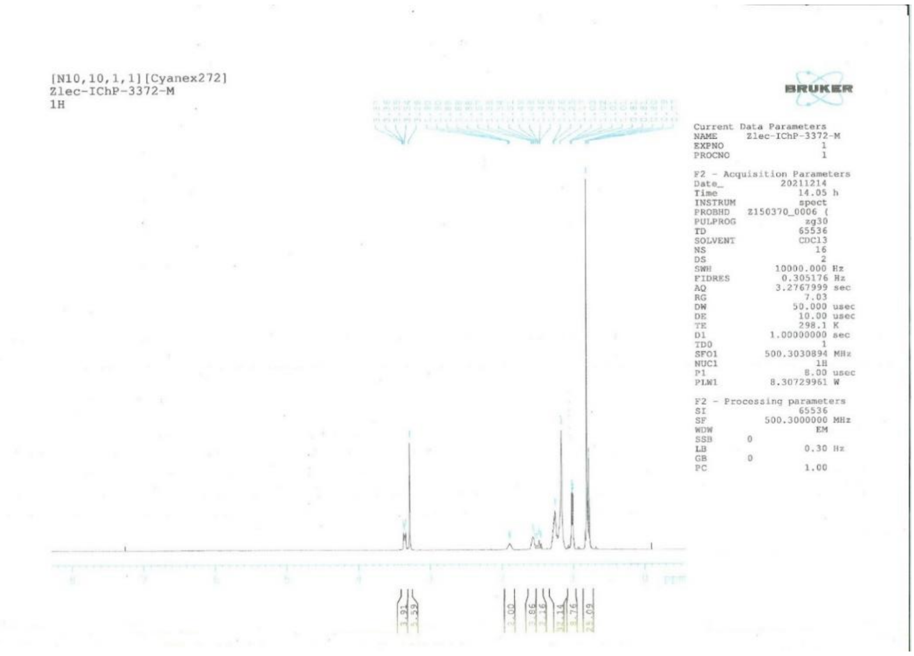

^{13}C NMR ($CDCl_3$, 125.8 MHz), δ: 14.00 (2 × CH_3), 22.54 (2 × CH_2), 22.66 (2 × CH_2), 24.26 (CH_3), 24.29 (CH_3), 24.31 (CH_3), 24.34 (CH_3), 25.40 (d, CH, J = 3.1 Hz), 25.46 (d, CH, J = 3,1 Hz), 26.24 (CH_2), 29.14 (2 × CH_2), 29.15 (2 × CH_2), 29.29 (CH_2), 29.32 (2 × CH_2), 30.23 (2 × CH_3), 30.23 (2 × CH_3), 31.14 (2 × C), 31.73 (2 × CH_2), 42.06 (d, 2 × **CH_2**[P=O]O-), J = 86.2 Hz), 51.19 (2 × **CH_3**[N^+]), 53.66 (CH_2), 53.73 (CH_2), 53.76 (CH_2), 53.83 (CH_2), 63.29 (2 × **CH_2**[N^+]) ppm.

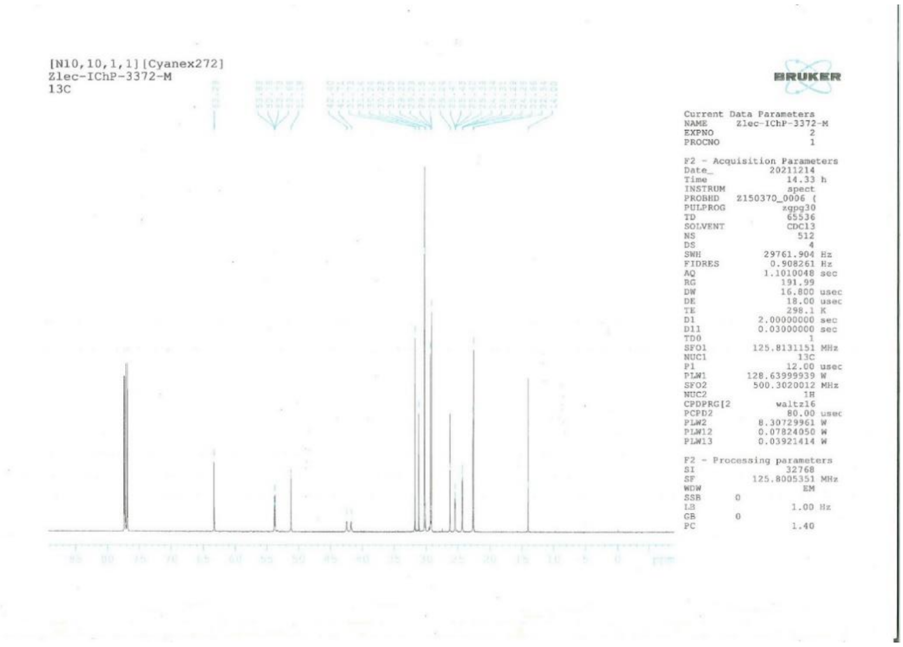

Supplement: Supplementary file 1 [file molecules-27-04984-s001.zip › molecules-1773590-supplementary.pdf]
